# Editorial: The relationship of oral and other body sites microbiome in human diseases

**DOI:** 10.3389/fcimb.2023.1276473

**Published:** 2023-08-23

**Authors:** Tiansong Xu, Ning Chen, Xuesong He, Feng Chen

**Affiliations:** ^1^ Central Laboratory, Peking University School and Hospital of Stomatology, Beijing, China; ^2^ Department of Obstetrics Department, Peking University People’s Hospital, Beijing, China; ^3^ Department of Microbiology, The Forsyth Institute, Cambridge, MA, United States; ^4^ Department of Oral Medicine, Infection and Immunity, Harvard School of Dental Medicine, Boston, MA, United States

**Keywords:** oral microbiome, interrelationship, other body sites microbiome, human diseases, extra-intestinal microbiome

The microbes colonize many regions of the human body, including the skin, the oral cavity, and the gastrointestinal, respiratory, and urogenital tracts. These host-associated microbial communities influence the ecological, evolutionary and immunological processes, and play a critical role in the host’s health and diseases (Hou et al., 2022).

The oral cavity is one of the most important interaction windows between the human body and the environment. As the second largest microbial community in the human body, the oral microbiota plays a vital role in supporting host health locally and systemically. Meanwhile, a dysbiotic oral microbiome may not only lead to oral diseases such as dental caries and periodontitis, but also contribute to extraoral conditions such as inflammatory bowel disease, atherosclerosis, arthritis, adverse pregnancy outcomes, and Alzheimer’s disease (Gao et al., 2018; Tuganbaev et al., 2022).

Since the dysbiotic state of the oral microbiome has been linked to the increased risk of many human disorders, it is important to achieve a better understanding of the physiological and pathological relationship of oral and other body sites microbiomes in human health and diseases (Lamont et al., 2018). The goal of the Research Topic was to explore the multifactorial factors that regulate the relationship and impact factors of the microbiomes of the oral cavity and other body sites for both research and clinical applications.

The association between oral and intestinal microbiota has been suggested in previous studies. Although being connected by the digestive tract, several defense mechanisms prevent ingested oral bacteria from colonizing the gut, such as bacterial clearance by gastric acid and pepsin in the stomach, and colonization exclusion by indigenous gut microbiome. However, when these defense mechanisms are compromised, oral microorganisms may disseminate to the gut and potentially disrupt intestinal homeostasis. Thus, it was necessary to understand how oral and gut microbiomes affect gastrointestinal tract ecology balance. Lu et al. reviewed possible entry pathways of the oral-gut microbiome axis, and the influence of oral microbes, on intestinal microbiota and the immune system. With the popularity of oral-gut microbes research, studying the influence of oral microbes on the intestine opens up a new perspective into preventing and managing intestinal diseases.

In another review article, Gershater et al. reviewed the characteristic of the oral microbiome of patients with cleft lip and palate (CL/P). The review shed light on the significant microbiota differences between CL/P patients and healthy individuals in different anatomic regions, including the teeth inside and adjacent to the cleft, oral cavity, nasal cavity, pharynx, and ear, as well as bodily fluids, secretions, and excretions. The study would be conducive to the advance of CL/P-specific microbiota treatment. Interestingly, the feature of a given species of CL/P patients and healthy individuals was not consistent from different sampling regions, suggesting that it must be done to sufficiently explore the non-typical bacterial and fungal species of CL/P patients in the future.

The oral microbiota may be a potential target for treating cardiovascular diseases (Tonelli et al., 2023). Hernández-Ruiz et al. evaluated oral microbiota diversity and circulating inflammatory profile in ST-segment elevation myocardial infarction (STEMI) patients stratified according to an inflammation-based risk scoring system and defined a non-causal link inferred between the cardiovascular risk of STEMI patients, and its relationship with the exacerbation of the inflammation. The study demonstrated that *Bacteriodetes* phylum was most abundant in STEMI patients, and *Prevotella* was the most abundant genus, with a higher proportion in periodontitis patients. *Prevotella* genus was found to correlate positively and significantly with elevated IL-6 concentration. The study emphasized the relationship between oral microbiome and circulatory system disease.

The balance of the microbial community is vital to infant health and development. Hou et al. developed a novel decontamination method for the new ultra-sensitive metagenomic sequencing method named 2bRAD-M and identified 11 and 8 potential biomarkers for neonatal bacterial meningitis in blood and cerebrospinal fluid (CSF). They further revealed 16 and 35 microbial species that were highly associated with the physiological indicators in blood and CSF, respectively. The study offered new microbiological markers to assist in the diagnosis of neonatal bacterial meningitis.

Also, Li et al. provided the future direction to apply the oral microbiome to improve human health. They depicted a synthetic and exhaustive landscape of focus and forefront in oral microbiome studies. The analysis suggested that the homeostatic balance of the oral microbiome, advanced microbial sequencing technology, connections with gut microbiota, and tumorigenesis have become hotspots in the aspect of the oral microbiome. Their results shed light on the increasing importance of the oral microbiome in the perspective of systemic diseases and states, more diverse fields such as immunity and cancer. Moreover, the study encouraged to prioritize fostering cooperation between institutions and researchers, both nationally and internationally.

## Conclusion

Evidence linking the oral microbiota to systemic diseases and overall health continues to accumulate. The physiological and pathological interaction between oral microbiome and microbiomes of other body sites has been suggested. A better understanding and effective management of oral microbiome-related diseases call for a holistic view of interactions among different host microbiota which requires systemic and multifactorial approaches.

## Author contributions

TX: Writing – original draft. NC: Supervision, Writing – review & editing. XH: Supervision, Writing – review & editing. FC: Supervision, Writing – review & editing.
